# Tricaesium tris­(pyridine-2,6-dicarboxyl­ato-κ^3^ *O* ^2^,*N*,*O* ^6^)lutetium(III) octa­hydrate

**DOI:** 10.1107/S1600536808029243

**Published:** 2008-09-20

**Authors:** Vincent Legrand, Flavien Aubert, Anthony D’Aléo, Philippe Rabiller, Olivier Maury

**Affiliations:** a Institut Laue Langevin, 6 rue Jules Horowitz, B.P.156, 38042 Grenoble Cedex 9, France; b Groupe Matiere Condensee et Materiaux, UMR CNRS 6626, Universite de Rennes 1, Campus de Beaulieu, 35042 Rennes cedex, France; c Laboratoire de Chimie, UMR CNRS 5182, ENS Lyon, 46 allee d’Italie, 69364 Lyon cedex 07, France

## Abstract

Colourless block crystals of the title compound, Cs_3_[Lu(dipic)_3_]·8H_2_O [dipic is dipicolinate or pyridine-2,6-dicarboxyl­ate, C_7_H_3_NO_4_] were synthesized by slow evaporation of the solvent. The crystal structure of this Lu^III^-complex, isostructural with the Dy^III^ and Eu^III^ complexes, was determined from a crystal twinned by inversion and consists of discrete [Lu(dipic)_3_]^3−^ anions, Cs^+^ cations and water mol­ecules involving hydrogen bonding. The Lu atom lies on a twofold rotation axis and is coordinated by six O atoms and three N atoms of three dipicolinate ligands. One Cs atom is also on a twofold axis. The unit cell can be regarded as successive layers along the crystallographic *c*-axis formed by [Lu(dipic)_3_]^3−^ anionic planes and [Cs^+^, H_2_O] cationic planes. In the crystal structure, although the H atoms attached to water mol­ecules could not be located, short O—O contacts clearly indicate the occurrence of an intricate hydrogen-bonded network through contacts with other water mol­ecules, Cs cations or with the O atoms of the dipicolinate ligands.

## Related literature

For potential applications of lanthanide complexes as second-order non-linear optical materials, see: Tancrez *et al.* (2005 ▶); Sénéchal *et al.* (2004 ▶). For the isostructural Eu^III^ complex, see: Brayshaw *et al.* (1995 ▶). For other related complexes, see: Murray *et al.* (1990 ▶). For related literature, see: Flack & Bernardinelli (1999 ▶, 2000 ▶).
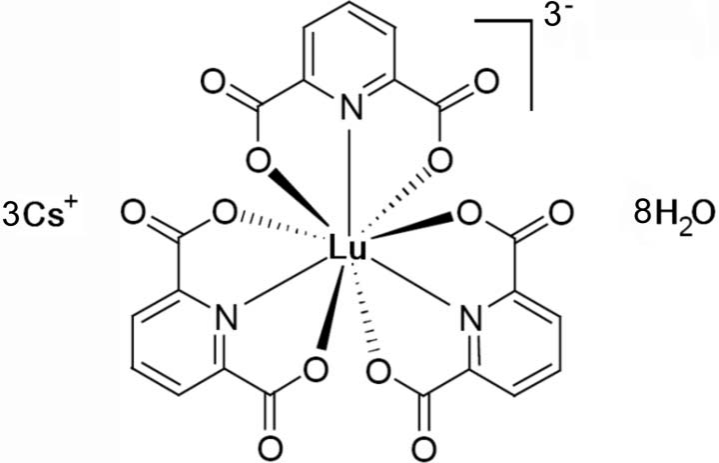


## Experimental

### 

#### Crystal data

Cs_3_[Lu(C_7_H_3_NO_4_)_3_]·8H_2_O
*M* *_r_* = 1213.14
Orthorhombic, 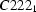

*a* = 10.0406 (2) Å
*b* = 17.8109 (6) Å
*c* = 18.4221 (5) Å
*V* = 3294.46 (16) Å^3^
*Z* = 4
Mo *K*α radiation
μ = 6.36 mm^−1^
*T* = 100 (2) K
0.20 × 0.19 × 0.19 mm

#### Data collection

Oxford Diffraction Xcalibur–Sapphire3 diffractometer
Absorption correction: Gaussian (*ABSORB*; DeTitta, 1985 ▶) *T*_min_ = 0.307, *T*_max_ = 0.425
51066 measured reflections
3520 independent reflections
3491 reflections with *I* > 2σ(*I*)
*R*_int_ = 0.045

#### Refinement

*R*[*F* ^2^ > 2σ(*F* ^2^)] = 0.025
*wR*(*F* ^2^) = 0.063
*S* = 1.46
3520 reflections
208 parameters
H-atom parameters constrained
Δρ_max_ = 3.00 e Å^−3^
Δρ_min_ = −0.94 e Å^−3^
Absolute structure: Flack (1983 ▶), 1501 Friedel pairs
Flack parameter: 0.270 (12)

### 

Data collection: *CrysAlis CCD* (Oxford Diffraction 2006 ▶); cell refinement: *CrysAlis RED* (Oxford Diffraction 2006 ▶); data reduction: *SORTAV* (Blessing, 1989 ▶); program(s) used to solve structure: *SIR97* (Altomare *et al.*, 1999 ▶); program(s) used to refine structure: *SHELXL97* (Sheldrick, 2008 ▶); molecular graphics: *ORTEP-3* (Farrugia, 1997 ▶); software used to prepare material for publication: *WinGX* (Farrugia, 1999 ▶).

## Supplementary Material

Crystal structure: contains datablocks I, global. DOI: 10.1107/S1600536808029243/dn2357sup1.cif

Structure factors: contains datablocks I. DOI: 10.1107/S1600536808029243/dn2357Isup2.hkl

Additional supplementary materials:  crystallographic information; 3D view; checkCIF report

## Figures and Tables

**Fig. 1.**
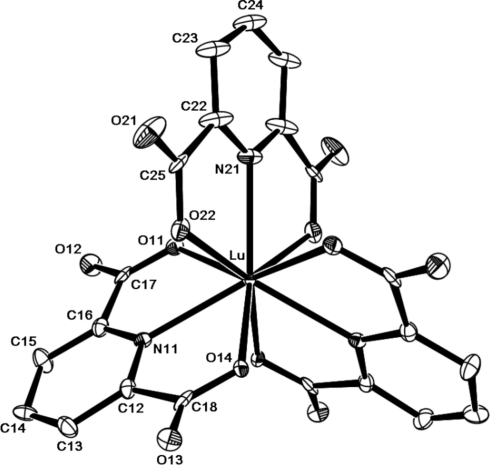
The molecular structure of [Lu(dipic)3]3- with the atom numbering scheme. Displacement ellipsoids are drawn at 50% probability level and H atoms are omitted for clarity.

**Fig. 2.**
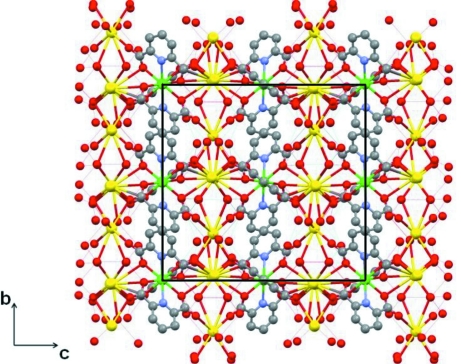
Crystal packing of Cs3[Lu(dipic)3].8H2O with hydrogen bonds (in dotted line) and coordinate bonds to caesium. [Lu(dipic)3]3- anions and Cs+ cations are distributed in the cell as successive anionic/cationic layers along the crystallographic c-axis (Lu atoms are in green, Cs in yellow, O in red, C in grey and N in blue).

## supplementary crystallographic information

### Comment

Lanthanide complexes attract considerable interest due to their magnetic and luminescent properties, but also for their potentialities in the field of second-order nonlinear optics (Sénéchal *et al.*, 2004; Tancrez *et al.*, 2005). Lanthanides emission can be sensitized either by direct absorption in the forbidden *f-f* transitions or *via* energy transfer from an organic ligand acting as antenna. Pulsed excitations induce long luminescence decay of the lanthanides and this time-gated emission can be used in medicine as probes in biological system for diagnosis or therapeutic purposes. In this context, lanthanide tris-dipicolinate (= pyridine-2,6-dicarboxylate) have been extensively studied both in solution and in solid state. We report the synthesis and structural characterization by X-ray diffraction measurements of the following complex: Cs_3_[Lu(dipic)_3_].8H_2_O, (I).

The compound (I) is isomorphous to the Eu^III^ complex (Brayshaw *et al.*, 1995). The asymmetric unit contains one Lu^III^ atom located on a twofold rotation axis, one and a half dipicolinate carboxylate ligand, two Cs^+^ cations and water molecules (Fig. 1). The unit cell could be regarded as successive layers along the crystallographic *c*-axis formed by [Lu(dipic)_3_]^3-^ anionic planes and [Cs^+^, H_2_O] cationic planes (Fig. 2).

Two types of Cs^+^ cations, which are bridged by both carboxylate and water oxygen atoms, form a chain extending throughout the crystal (Fig. 2). The chain could be considered as a sequence of ten-coordinate caesium ions linked, through bridging coordination, to eight-coordinate caesium ions. All water molecules are involved in coordination either to Cs^+^, to other water molecules or to the oxygen atoms of the dipicolinate ligands.

The Lu^III^ atom, being nine-coordinated by six O and three N atoms of three dipicolinate ligands, is in the centre of a quite regular tricapped trigonal prismatic coordination sphere (Fig. 1). In those compounds, metal-to-metal distances play an important role concerning the electronic interactions affecting luminescence behaviour. For (I) the shortest Lu···Lu separation is 10.22 Å in contrast with the 4.50Å of the shortest Cs···Lu separation. These distances compare well with those observed in the Cs_3_[Eu(dipic)_3_].9H_2_O (Brayshaw *et al.*, 1995). Geometrical parameters of the dipicolinate ligand are found to be in agreement with those of other structures containing dipicolinate.

Although the H atoms attached to water molecules could not be located, short O—O contacts clearly indicate the occurrence of a complicated hydrogen-bonded network. The water molecules are positioned in a channel formed by the successive anionic and cationic layers. The water molecules in this channel appear to have considerable freedom of motion. The structural refinement of (I) reveals high values of the thermal motion parameters of the water oxygen atoms indicating that most of the water molecules are incorporated in the unit cell in disordered positions. This effect was also observed in the complexes Cs_3_[Eu(dipic)_3_].9H_2_O, [Co(sar)][Lu(dipic)_3_].13H_2_O and Na_3_[Eu(dipic)_3_].nH_2_O (Brayshaw *et al.*, 1995; Murray *et al.*, 1990) which have also been investigated by emission spectroscopy, showing that different arrangements of the water molecules induce different crystal field splittings, clearly pointed out in the emission spectra.

### Experimental

The caesium salt of the tris(dipicolinato)-Lu^III^ anion was prepared by reaction between dipicolinic acid (3Eq) and CsCO_3_ acting as base and counter-anion in water. After stirring until everything dissolved, LuCl_3_.6H_2_O (1Eq) was added and the reaction was stirred at room temperature for two more hours. The water was evaporated and the white solid was dissolved in the minimum of boiling water. The complex was purified by three successive crystallizations at 4°C.

### Refinement

H atoms of the dipic ligand were placed in geometrically idealized positions with fixed C—H distances (0.93 Å) and refined in riding mode, with *U_iso_*(H) = 1.2*U_eq_*(C). Due to the large disorder observed on the water molecules, it was not possible to position correctly the associated H atoms which were not included in the final refinement.

The crystal structure of (I) indicates a disorder of the O4w water oxygen atom which was refined at two independent positions; the two O4w positions have complementary refined occupancies of 0.69 (2) and 0.31 (2) for the major and minor positions respectively. The highest peak in the final difference Fourier map is located at 2.26 Å from O4wA and stands on a two fold axis lying equidistant to 3 heavy atoms. *PLATON* shows that there are no accessible voids in the cell and so this position might be related to the occurrence of diffraction ripple from the 3 heavy atoms. The deepest hole is at 1.20 Å from atom Lu1. The atom C24, which stands on a symmetry axis, lies essentially equidistant from two heavy atoms and stands on a diffraction ripple lying with a maximum right beside the C24 site; the thermal motion parameters of C24 were so constraint to be identical as C22 and C23.

The absolute structure parameter was calculated using *SHELX97* (Sheldrick, 2008). Owing to the occurrence of strong inversion-distinguishing power (Flack, 1983; Flack and Bernardinelli, 1999; 2000), the value given for the Flack parameter and its standard uncertainty (0.270 (12)) could be regarded as reliable, and then indicates that the crystal is twinned by inversion (Brayshaw *et al., *1995).

### Crystal data

**Table:**

Cs_3_[Lu(C_7_H_3_NO_4_)_3_]·8H_2_O	*F*(000) = 2312
*M**_r_* = 1213.14	*D*_x_ = 2.446 Mg m^−^^3^
Orthorhombic, *C*222_1_	Mo *K*α radiation, λ = 0.71073 Å
Hall symbol: C 2c 2	Cell parameters from 17659 reflections
*a* = 10.0406 (2) Å	θ = 3.2–59.9°
*b* = 17.8109 (6) Å	µ = 6.36 mm^−^^1^
*c* = 18.4221 (5) Å	*T* = 100 K
*V* = 3294.46 (16) Å^3^	Block, colourless
*Z* = 4	0.20 × 0.19 × 0.19 mm

### Data collection

**Table:**

Oxford Diffraction Xcalibur-Sapphire3 diffractometer	3520 independent reflections
Radiation source: fine-focus sealed tube	3491 reflections with *I* > 2σ(*I*)
graphite	*R*_int_ = 0.045
ω scans	θ_max_ = 27.0°, θ_min_ = 3.2°
Absorption correction: gaussian (ABSORB; DeTitta, 1985)	*h* = −12→12
*T*_min_ = 0.308, *T*_max_ = 0.425	*k* = −22→22
51066 measured reflections	*l* = −23→23

### Refinement

**Table:**

Refinement on *F*^2^	Secondary atom site location: difference Fourier map
Least-squares matrix: full	Hydrogen site location: inferred from neighbouring sites
*R*[*F*^2^ > 2σ(*F*^2^)] = 0.025	H-atom parameters constrained
*wR*(*F*^2^) = 0.063	*w* = 1/[σ^2^(*F*_o_^2^) + (0.0149*P*)^2^ + 27.044*P*] where *P* = (*F*_o_^2^ + 2*F*_c_^2^)/3
*S* = 1.46	(Δ/σ)_max_ < 0.001
3520 reflections	Δρ_max_ = 3.00 e Å^−^^3^
208 parameters	Δρ_min_ = −0.94 e Å^−^^3^
0 restraints	Absolute structure: Flack (1983),1501 Friedel pairs
Primary atom site location: structure-invariant direct methods	Flack parameter: 0.270 (12)

### Special details

**Table:**

Geometry. All e.s.d.'s (except the e.s.d. in the dihedral angle between two l.s. planes) are estimated using the full covariance matrix. The cell e.s.d.'s are taken into account individually in the estimation of e.s.d.'s in distances, angles and torsion angles; correlations between e.s.d.'s in cell parameters are only used when they are defined by crystal symmetry. An approximate (isotropic) treatment of cell e.s.d.'s is used for estimating e.s.d.'s involving l.s. planes.
Refinement. Refinement of *F*^2^ against ALL reflections. The weighted *R*-factor *wR* and goodness of fit *S* are based on *F*^2^, conventional *R*-factors *R* are based on *F*, with *F* set to zero for negative *F*^2^. The threshold expression of *F*^2^ > σ(*F*^2^) is used only for calculating *R*-factors(gt) *etc*. and is not relevant to the choice of reflections for refinement. *R*-factors based on *F*^2^ are statistically about twice as large as those based on *F*, and *R*- factors based on ALL data will be even larger.

### Fractional atomic coordinates and isotropic or equivalent isotropic displacement parameters (Å^2^)

**Table:**

	*x*	*y*	*z*	*U*_iso_*/*U*_eq_	Occ. (<1)
Lu1	0.75054 (5)	0.0000	0.0000	0.00950 (7)	
Cs1	0.74407 (4)	0.021678 (17)	−0.24335 (2)	0.01702 (8)	
Cs2	0.5000	−0.26389 (3)	−0.2500	0.01796 (11)	
N11	0.8713 (4)	−0.1197 (2)	−0.0042 (3)	0.0107 (8)	
O11	0.6846 (4)	−0.0835 (2)	−0.0950 (2)	0.0149 (8)	
O12	0.7226 (5)	−0.1846 (3)	−0.1649 (2)	0.0228 (12)	
O13	1.0389 (5)	−0.0907 (3)	0.1601 (2)	0.0174 (11)	
O14	0.9174 (4)	−0.01382 (19)	0.0906 (2)	0.0124 (8)	
C12	0.9612 (6)	−0.1355 (3)	0.0466 (3)	0.0131 (12)	
C13	1.0349 (6)	−0.2016 (3)	0.0468 (3)	0.0187 (13)	
H13	1.1001	−0.2089	0.0819	0.022*	
C14	1.0140 (14)	−0.2518 (3)	−0.0006 (8)	0.040 (2)	
H14	1.0567	−0.2980	0.0026	0.048*	
C15	0.9185 (7)	−0.2357 (4)	−0.0624 (3)	0.0199 (13)	
H15	0.9073	−0.2688	−0.1010	0.024*	
C16	0.8496 (6)	−0.1693 (3)	−0.0580 (3)	0.0132 (11)	
C17	0.7438 (8)	−0.1440 (3)	−0.1112 (3)	0.0146 (6)	
C18	0.9756 (6)	−0.0754 (3)	0.1041 (4)	0.0146 (6)	
N21	0.5071 (8)	0.0000	0.0000	0.0173 (12)	
O21	0.4794 (7)	−0.1104 (3)	0.1573 (3)	0.0323 (15)	
O22	0.6543 (5)	−0.0785 (2)	0.0880 (2)	0.0168 (9)	
C22	0.4415 (7)	−0.0390 (4)	0.0490 (3)	0.0242 (9)	
C23	0.3047 (7)	−0.0426 (4)	0.0509 (3)	0.0242 (9)	
H23	0.2584	−0.0719	0.0843	0.029*	
C24	0.2430 (13)	0.0000	0.0000	0.0242 (9)	
H24	0.1503	0.0000	0.0000	0.029*	
C25	0.5310 (6)	−0.0805 (4)	0.1040 (4)	0.0146 (6)	
O1W	0.7467 (9)	−0.1739 (4)	−0.3818 (4)	0.0584 (17)	
O2W	0.9514 (7)	−0.0907 (4)	−0.3228 (4)	0.0424 (18)	
O3W	0.5456 (5)	−0.0888 (3)	−0.3207 (3)	0.0240 (12)	
O4WA	0.6970 (8)	0.2031 (4)	−0.2207 (6)	0.032 (3)	0.69 (2)
O4WB	0.7228 (15)	0.1956 (8)	−0.1715 (11)	0.023 (6)	0.31 (2)

### Atomic displacement parameters (Å^2^)

**Table:**

	*U*^11^	*U*^22^	*U*^33^	*U*^12^	*U*^13^	*U*^23^
Lu1	0.00740 (14)	0.01013 (12)	0.01097 (13)	0.000	0.000	−0.00081 (10)
Cs1	0.01540 (16)	0.02237 (15)	0.01327 (15)	−0.00244 (13)	0.00109 (19)	−0.00051 (11)
Cs2	0.0245 (3)	0.01430 (19)	0.0151 (2)	0.000	−0.0028 (2)	0.000
N11	0.008 (2)	0.0127 (18)	0.011 (2)	−0.0017 (15)	0.0012 (18)	0.0004 (17)
O11	0.013 (2)	0.013 (2)	0.018 (2)	−0.0015 (16)	−0.0019 (16)	−0.0004 (16)
O12	0.023 (4)	0.023 (2)	0.022 (2)	0.0013 (18)	−0.0084 (19)	−0.0097 (17)
O13	0.019 (3)	0.023 (3)	0.010 (2)	0.0014 (18)	−0.0035 (16)	0.0022 (17)
O14	0.010 (2)	0.008 (2)	0.018 (2)	0.0010 (14)	−0.0021 (15)	−0.0017 (14)
C12	0.015 (3)	0.011 (2)	0.014 (3)	−0.0009 (19)	0.001 (2)	0.002 (2)
C13	0.018 (4)	0.017 (3)	0.021 (3)	0.004 (2)	−0.004 (2)	0.001 (2)
C14	0.033 (6)	0.023 (3)	0.066 (5)	−0.003 (3)	−0.040 (5)	0.009 (3)
C15	0.026 (4)	0.018 (3)	0.016 (3)	0.007 (3)	−0.002 (2)	−0.005 (2)
C16	0.015 (3)	0.012 (3)	0.012 (3)	−0.002 (2)	0.003 (2)	0.001 (2)
C17	0.0135 (16)	0.0138 (13)	0.0166 (13)	−0.0096 (16)	0.0027 (15)	−0.0028 (10)
C18	0.0135 (16)	0.0138 (13)	0.0166 (13)	−0.0096 (16)	0.0027 (15)	−0.0028 (10)
N21	0.009 (3)	0.027 (3)	0.016 (3)	0.000	0.000	−0.010 (3)
O21	0.038 (4)	0.039 (3)	0.021 (2)	−0.021 (3)	0.014 (2)	−0.009 (2)
O22	0.020 (3)	0.013 (2)	0.0168 (19)	−0.0027 (17)	0.0040 (17)	−0.0020 (15)
C22	0.0106 (19)	0.048 (2)	0.0143 (17)	−0.007 (2)	0.0024 (15)	−0.0146 (17)
C23	0.0106 (19)	0.048 (2)	0.0143 (17)	−0.007 (2)	0.0024 (15)	−0.0146 (17)
C24	0.0106 (19)	0.048 (2)	0.0143 (17)	−0.007 (2)	0.0024 (15)	−0.0146 (17)
C25	0.0135 (16)	0.0138 (13)	0.0166 (13)	−0.0096 (16)	0.0027 (15)	−0.0028 (10)
O1W	0.046 (4)	0.079 (5)	0.051 (3)	0.016 (5)	−0.002 (4)	−0.012 (3)
O2W	0.047 (4)	0.021 (3)	0.059 (4)	0.003 (3)	0.028 (3)	0.001 (3)
O3W	0.018 (3)	0.032 (3)	0.022 (3)	−0.004 (2)	−0.005 (2)	0.004 (2)
O4WA	0.028 (4)	0.030 (4)	0.039 (6)	−0.002 (3)	−0.003 (3)	0.007 (3)
O4WB	0.012 (11)	0.018 (7)	0.038 (13)	0.005 (5)	0.011 (7)	0.015 (6)

### Geometric parameters (Å, °)

**Table:**

Lu1—O22^i^	2.348 (4)	O11—C17	1.267 (8)
Lu1—O22	2.348 (4)	O12—C17	1.244 (7)
Lu1—O14	2.377 (4)	O13—C18	1.242 (9)
Lu1—O14^i^	2.377 (4)	O13—Cs1^ix^	3.070 (5)
Lu1—O11	2.390 (4)	O13—Cs2^x^	3.100 (5)
Lu1—O11^i^	2.390 (4)	O13—Cs1^i^	3.553 (5)
Lu1—N21	2.445 (8)	O14—C18	1.267 (8)
Lu1—N11^i^	2.454 (4)	O14—Cs1^i^	3.312 (4)
Lu1—N11	2.454 (4)	C12—C13	1.390 (8)
Lu1—Cs1	4.5002 (4)	C12—C18	1.513 (8)
Lu1—Cs1^i^	4.5002 (4)	C13—C14	1.267 (13)
Cs1—O13^ii^	3.070 (5)	C13—Cs2^x^	3.810 (6)
Cs1—O3W	3.142 (5)	C13—H13	0.9300
Cs1—O22^i^	3.167 (4)	C14—C15	1.517 (12)
Cs1—O2W	3.237 (6)	C14—H14	0.9300
Cs1—O4WA	3.293 (8)	C15—C16	1.373 (9)
Cs1—O21^iii^	3.299 (6)	C15—H15	0.9300
Cs1—O14^i^	3.312 (4)	C16—C17	1.514 (9)
Cs1—O11	3.367 (4)	C18—Cs1^i^	3.592 (6)
Cs1—O4WB	3.375 (16)	N21—C22^i^	1.315 (8)
Cs1—O21^i^	3.474 (6)	N21—C22	1.315 (8)
Cs1—C25^i^	3.502 (6)	O21—C25	1.230 (9)
Cs1—O13^i^	3.553 (5)	O21—Cs1^xi^	3.299 (6)
Cs2—O12	3.073 (5)	O21—Cs1^i^	3.474 (6)
Cs2—O12^iv^	3.073 (5)	O22—C25	1.273 (8)
Cs2—O13^v^	3.100 (5)	O22—Cs1^i^	3.167 (4)
Cs2—O13^vi^	3.100 (5)	C22—C23	1.376 (10)
Cs2—O4WA^vii^	3.145 (8)	C22—C25	1.544 (10)
Cs2—O4WA^viii^	3.145 (8)	C23—C24	1.356 (9)
Cs2—O4WB^vii^	3.219 (14)	C23—Cs1^xi^	3.840 (6)
Cs2—O4WB^viii^	3.219 (14)	C23—H23	0.9300
Cs2—O3W	3.410 (6)	C24—C23^i^	1.356 (9)
Cs2—O3W^iv^	3.410 (6)	C24—H24	0.9300
Cs2—C13^v^	3.810 (6)	C25—Cs1^i^	3.502 (6)
Cs2—C13^vi^	3.810 (6)	O3W—Cs1^iv^	3.705 (6)
N11—C12	1.330 (7)	O4WA—Cs2^xii^	3.145 (8)
N11—C16	1.345 (7)	O4WB—Cs2^xii^	3.219 (14)
			
O22^i^—Lu1—O22	131.4 (2)	O13^v^—Cs2—O4WA^viii^	82.77 (19)
O22^i^—Lu1—O14	146.72 (14)	O13^vi^—Cs2—O4WA^viii^	79.26 (18)
O22—Lu1—O14	75.16 (15)	O4WA^vii^—Cs2—O4WA^viii^	158.5 (3)
O22^i^—Lu1—O14^i^	75.16 (15)	O12—Cs2—O4WB^vii^	120.2 (4)
O22—Lu1—O14^i^	146.72 (14)	O12^iv^—Cs2—O4WB^vii^	72.8 (3)
O14—Lu1—O14^i^	90.4 (2)	O13^v^—Cs2—O4WB^vii^	86.8 (3)
O22^i^—Lu1—O11	75.58 (13)	O13^vi^—Cs2—O4WB^vii^	71.4 (3)
O22—Lu1—O11	91.20 (14)	O4WA^vii^—Cs2—O4WB^vii^	17.2 (3)
O14—Lu1—O11	130.16 (14)	O4WA^viii^—Cs2—O4WB^vii^	150.6 (3)
O14^i^—Lu1—O11	75.28 (14)	O12—Cs2—O4WB^viii^	72.8 (3)
O22^i^—Lu1—O11^i^	91.20 (14)	O12^iv^—Cs2—O4WB^viii^	120.2 (4)
O22—Lu1—O11^i^	75.58 (13)	O13^v^—Cs2—O4WB^viii^	71.4 (3)
O14—Lu1—O11^i^	75.28 (14)	O13^vi^—Cs2—O4WB^viii^	86.8 (3)
O14^i^—Lu1—O11^i^	130.16 (14)	O4WA^vii^—Cs2—O4WB^viii^	150.6 (3)
O11—Lu1—O11^i^	147.8 (2)	O4WA^viii^—Cs2—O4WB^viii^	17.2 (3)
O22^i^—Lu1—N21	65.71 (12)	O4WB^vii^—Cs2—O4WB^viii^	154.1 (5)
O22—Lu1—N21	65.71 (12)	O12—Cs2—O3W	71.14 (12)
O14—Lu1—N21	134.81 (10)	O12^iv^—Cs2—O3W	58.84 (12)
O14^i^—Lu1—N21	134.81 (10)	O13^v^—Cs2—O3W	125.24 (14)
O11—Lu1—N21	73.90 (11)	O13^vi^—Cs2—O3W	162.07 (13)
O11^i^—Lu1—N21	73.90 (11)	O4WA^vii^—Cs2—O3W	111.48 (18)
O22^i^—Lu1—N11^i^	73.00 (16)	O4WA^viii^—Cs2—O3W	88.59 (17)
O22—Lu1—N11^i^	134.33 (16)	O4WB^vii^—Cs2—O3W	119.5 (3)
O14—Lu1—N11^i^	73.72 (14)	O4WB^viii^—Cs2—O3W	85.3 (3)
O14^i^—Lu1—N11^i^	65.42 (14)	O12—Cs2—O3W^iv^	58.84 (12)
O11—Lu1—N11^i^	134.47 (16)	O12^iv^—Cs2—O3W^iv^	71.14 (12)
O11^i^—Lu1—N11^i^	64.75 (15)	O13^v^—Cs2—O3W^iv^	162.07 (13)
N21—Lu1—N11^i^	119.60 (10)	O13^vi^—Cs2—O3W^iv^	125.24 (14)
O22^i^—Lu1—N11	134.33 (16)	O4WA^vii^—Cs2—O3W^iv^	88.59 (17)
O22—Lu1—N11	73.00 (16)	O4WA^viii^—Cs2—O3W^iv^	111.48 (19)
O14—Lu1—N11	65.42 (14)	O4WB^vii^—Cs2—O3W^iv^	85.3 (3)
O14^i^—Lu1—N11	73.72 (14)	O4WB^viii^—Cs2—O3W^iv^	119.5 (3)
O11—Lu1—N11	64.75 (15)	O3W—Cs2—O3W^iv^	47.76 (19)
O11^i^—Lu1—N11	134.47 (16)	O12—Cs2—C13^v^	129.96 (13)
N21—Lu1—N11	119.60 (10)	O12^iv^—Cs2—C13^v^	60.42 (13)
N11^i^—Lu1—N11	120.8 (2)	O13^v^—Cs2—C13^v^	47.80 (13)
O22^i^—Lu1—Cs1	41.88 (11)	O13^vi^—Cs2—C13^v^	113.68 (13)
O22—Lu1—Cs1	137.11 (11)	O4WA^vii^—Cs2—C13^v^	92.8 (2)
O14—Lu1—Cs1	135.91 (10)	O4WA^viii^—Cs2—C13^v^	83.7 (2)
O14^i^—Lu1—Cs1	45.74 (10)	O4WB^vii^—Cs2—C13^v^	109.0 (4)
O11—Lu1—Cs1	47.15 (10)	O4WB^viii^—Cs2—C13^v^	66.5 (4)
O11^i^—Lu1—Cs1	132.23 (10)	O3W—Cs2—C13^v^	77.57 (13)
N21—Lu1—Cs1	89.173 (8)	O3W^iv^—Cs2—C13^v^	120.70 (13)
N11^i^—Lu1—Cs1	87.94 (12)	O12—Cs2—C13^vi^	60.42 (13)
N11—Lu1—Cs1	92.88 (12)	O12^iv^—Cs2—C13^vi^	129.96 (13)
O22^i^—Lu1—Cs1^i^	137.11 (11)	O13^v^—Cs2—C13^vi^	113.68 (13)
O22—Lu1—Cs1^i^	41.88 (11)	O13^vi^—Cs2—C13^vi^	47.80 (13)
O14—Lu1—Cs1^i^	45.74 (10)	O4WA^vii^—Cs2—C13^vi^	83.7 (2)
O14^i^—Lu1—Cs1^i^	135.91 (10)	O4WA^viii^—Cs2—C13^vi^	92.8 (2)
O11—Lu1—Cs1^i^	132.23 (10)	O4WB^vii^—Cs2—C13^vi^	66.5 (4)
O11^i^—Lu1—Cs1^i^	47.15 (10)	O4WB^viii^—Cs2—C13^vi^	109.0 (4)
N21—Lu1—Cs1^i^	89.173 (8)	O3W—Cs2—C13^vi^	120.70 (13)
N11^i^—Lu1—Cs1^i^	92.88 (12)	O3W^iv^—Cs2—C13^vi^	77.57 (13)
N11—Lu1—Cs1^i^	87.94 (12)	C13^v^—Cs2—C13^vi^	161.41 (18)
Cs1—Lu1—Cs1^i^	178.347 (17)	C12—N11—C16	119.3 (5)
O13^ii^—Cs1—O3W	115.98 (14)	C12—N11—Lu1	119.8 (4)
O13^ii^—Cs1—O22^i^	126.72 (12)	C16—N11—Lu1	120.9 (4)
O3W—Cs1—O22^i^	115.44 (13)	C17—O11—Lu1	124.9 (4)
O13^ii^—Cs1—O2W	61.89 (12)	C17—O11—Cs1	101.4 (3)
O3W—Cs1—O2W	79.37 (13)	Lu1—O11—Cs1	101.50 (14)
O22^i^—Cs1—O2W	142.10 (16)	C17—O12—Cs2	142.9 (5)
O13^ii^—Cs1—O4WA	77.43 (18)	C18—O13—Cs1^ix^	139.2 (4)
O3W—Cs1—O4WA	125.53 (19)	C18—O13—Cs2^x^	124.2 (4)
O22^i^—Cs1—O4WA	62.0 (2)	Cs1^ix^—O13—Cs2^x^	96.56 (13)
O2W—Cs1—O4WA	139.14 (19)	C18—O13—Cs1^i^	81.8 (4)
O13^ii^—Cs1—O21^iii^	88.27 (12)	Cs1^ix^—O13—Cs1^i^	101.70 (14)
O3W—Cs1—O21^iii^	67.47 (13)	Cs2^x^—O13—Cs1^i^	87.33 (12)
O22^i^—Cs1—O21^iii^	98.92 (14)	C18—O14—Lu1	123.5 (4)
O2W—Cs1—O21^iii^	118.85 (19)	C18—O14—Cs1^i^	92.2 (4)
O4WA—Cs1—O21^iii^	60.2 (2)	Lu1—O14—Cs1^i^	103.33 (13)
O13^ii^—Cs1—O14^i^	97.84 (11)	N11—C12—C13	122.8 (5)
O3W—Cs1—O14^i^	133.79 (12)	N11—C12—C18	114.0 (5)
O22^i^—Cs1—O14^i^	52.77 (10)	C13—C12—C18	123.1 (5)
O2W—Cs1—O14^i^	91.16 (16)	C14—C13—C12	120.5 (7)
O4WA—Cs1—O14^i^	90.54 (19)	C14—C13—Cs2^x^	123.1 (7)
O21^iii^—Cs1—O14^i^	148.13 (12)	C12—C13—Cs2^x^	95.2 (4)
O13^ii^—Cs1—O11	144.99 (12)	C14—C13—H13	119.7
O3W—Cs1—O11	84.68 (12)	C12—C13—H13	119.7
O22^i^—Cs1—O11	52.66 (10)	Cs2^x^—C13—H13	50.1
O2W—Cs1—O11	97.89 (14)	C13—C14—C15	119.3 (7)
O4WA—Cs1—O11	114.7 (2)	C13—C14—H14	120.4
O21^iii^—Cs1—O11	126.59 (13)	C15—C14—H14	120.4
O14^i^—Cs1—O11	51.67 (10)	C16—C15—C14	115.9 (6)
O13^ii^—Cs1—O4WB	84.6 (3)	C16—C15—H15	122.1
O3W—Cs1—O4WB	135.4 (3)	C14—C15—H15	122.1
O22^i^—Cs1—O4WB	48.2 (3)	N11—C16—C15	121.9 (5)
O2W—Cs1—O4WB	141.8 (3)	N11—C16—C17	113.3 (5)
O4WA—Cs1—O4WB	16.4 (3)	C15—C16—C17	124.8 (5)
O21^iii^—Cs1—O4WB	74.7 (4)	O12—C17—O11	127.0 (7)
O14^i^—Cs1—O4WB	74.8 (4)	O12—C17—C16	117.5 (6)
O11—Cs1—O4WB	100.4 (3)	O11—C17—C16	115.5 (5)
O13^ii^—Cs1—O21^i^	128.72 (14)	O12—C17—Cs1	86.7 (3)
O3W—Cs1—O21^i^	90.39 (15)	O11—C17—Cs1	59.6 (3)
O22^i^—Cs1—O21^i^	39.15 (12)	C16—C17—Cs1	129.9 (4)
O2W—Cs1—O21^i^	168.30 (16)	O13—C18—O14	126.1 (6)
O4WA—Cs1—O21^i^	52.14 (18)	O13—C18—C12	118.4 (6)
O21^iii^—Cs1—O21^i^	61.01 (17)	O14—C18—C12	115.5 (5)
O14^i^—Cs1—O21^i^	91.90 (11)	O13—C18—Cs1^i^	78.2 (4)
O11—Cs1—O21^i^	75.32 (12)	O14—C18—Cs1^i^	67.2 (3)
O4WB—Cs1—O21^i^	49.9 (3)	C12—C18—Cs1^i^	128.8 (4)
O13^ii^—Cs1—C25^i^	137.62 (15)	C22^i^—N21—C22	119.9 (9)
O3W—Cs1—C25^i^	97.62 (14)	C22^i^—N21—Lu1	120.0 (4)
O22^i^—Cs1—C25^i^	21.25 (13)	C22—N21—Lu1	120.0 (4)
O2W—Cs1—C25^i^	155.39 (16)	C25—O21—Cs1^xi^	121.5 (5)
O4WA—Cs1—C25^i^	61.7 (2)	C25—O21—Cs1^i^	81.1 (4)
O21^iii^—Cs1—C25^i^	81.26 (15)	Cs1^xi^—O21—Cs1^i^	92.82 (13)
O14^i^—Cs1—C25^i^	73.18 (12)	C25—O22—Lu1	125.2 (4)
O11—Cs1—C25^i^	57.51 (13)	C25—O22—Cs1^i^	94.4 (4)
O4WB—Cs1—C25^i^	53.1 (3)	Lu1—O22—Cs1^i^	108.45 (15)
O21^i^—Cs1—C25^i^	20.31 (14)	N21—C22—C23	122.8 (7)
O13^ii^—Cs1—O13^i^	61.34 (14)	N21—C22—C25	114.3 (6)
O3W—Cs1—O13^i^	160.15 (13)	C23—C22—C25	122.8 (6)
O22^i^—Cs1—O13^i^	74.71 (11)	C24—C23—C22	114.3 (8)
O2W—Cs1—O13^i^	82.72 (16)	C24—C23—Cs1^xi^	124.7 (4)
O4WA—Cs1—O13^i^	74.07 (18)	C22—C23—Cs1^xi^	98.5 (4)
O21^iii^—Cs1—O13^i^	129.85 (12)	C24—C23—H23	122.8
O14^i^—Cs1—O13^i^	37.82 (10)	C22—C23—H23	122.8
O11—Cs1—O13^i^	89.41 (11)	Cs1^xi^—C23—H23	48.6
O4WB—Cs1—O13^i^	64.3 (3)	C23^i^—C24—C23	125.6 (11)
O21^i^—Cs1—O13^i^	106.43 (12)	C23^i^—C24—H24	117.2
C25^i^—Cs1—O13^i^	95.10 (14)	C23—C24—H24	117.2
O12—Cs2—O12^iv^	125.26 (18)	O21—C25—O22	127.3 (7)
O12—Cs2—O13^v^	138.44 (13)	O21—C25—C22	119.0 (6)
O12^iv^—Cs2—O13^v^	91.16 (13)	O22—C25—C22	113.6 (5)
O12—Cs2—O13^vi^	91.16 (13)	O21—C25—Cs1^i^	78.6 (4)
O12^iv^—Cs2—O13^vi^	138.44 (13)	O22—C25—Cs1^i^	64.4 (3)
O13^v^—Cs2—O13^vi^	66.60 (18)	C22—C25—Cs1^i^	133.9 (4)
O12—Cs2—O4WA^vii^	134.6 (2)	Cs1—O3W—Cs2	119.00 (16)
O12^iv^—Cs2—O4WA^vii^	58.0 (2)	Cs1—O3W—Cs1^iv^	91.19 (14)
O13^v^—Cs2—O4WA^vii^	79.26 (18)	Cs2—O3W—Cs1^iv^	104.99 (14)
O13^vi^—Cs2—O4WA^vii^	82.77 (19)	Cs2^xii^—O4WA—Cs1	91.3 (2)
O12—Cs2—O4WA^viii^	58.0 (2)	Cs2^xii^—O4WB—Cs1	88.6 (4)
O12^iv^—Cs2—O4WA^viii^	134.6 (2)		

Symmetry codes: (i) *x*, −*y*, −*z*; (ii) −*x*+2, −*y*, *z*−1/2; (iii) −*x*+1, −*y*, *z*−1/2; (iv) −*x*+1, *y*, −*z*−1/2; (v) −*x*+3/2, −*y*−1/2, *z*−1/2; (vi) *x*−1/2, −*y*−1/2, −*z*; (vii) *x*−1/2, *y*−1/2, *z*; (viii) −*x*+3/2, *y*−1/2, −*z*−1/2; (ix) −*x*+2, −*y*, *z*+1/2; (x) −*x*+3/2, −*y*−1/2, *z*+1/2; (xi) −*x*+1, −*y*, *z*+1/2; (xii) *x*+1/2, *y*+1/2, *z*.
